# A Critical Analysis of the Clinical Use of Incretin-Based Therapies: Efficacy and Adverse Events

**DOI:** 10.26502/acmcr.96550740

**Published:** 2025-12-30

**Authors:** Ilana Radparvar, Devendra K. Agrawal

**Affiliations:** Department of Translational Research, College of Osteopathic Medicine of the Pacific, Western University of Health Sciences, Pomona, California 91766 USA

**Keywords:** Adverse effects, Cardiometabolic disease, GLP-1 Receptor agonists, Incretin hormones, Obesity, semaglutide, Tirzepatide, Type 2 diabetes, Weight loss therapy

## Abstract

Obesity is a major public health challenge in the United States, despite widespread implementation of regulated diet plans, exercise programs, behavioral interventions, and surgical procedures. The emergence of incretin-based therapies, particularly GLP-1 (glucagon-like peptide-1) and GIP (glucose-dependent insulinotropic polypeptide) receptor agonists, has transformed the therapeutic landscape for initially type 2 diabetes and now obesity. Beyond their metabolic effects, incretin therapies exert meaningful cardiovascular, gastrointestinal, and neuroprotective actions. Semaglutide, a GLP-1 receptor agonist, and tripeptide, a dual GLP-1/GIP agonist, demonstrate substantial weight reduction, improved glycemic control, and reductions in cardiometabolic risk factors. Tirzepatide consistently produces greater weight loss effects than semaglutide, likely due to synergistic dual-receptor effects. However, these therapies are accompanied by adverse effects, most commonly gastrointestinal disturbances, and, less frequently, gallbladder disease, pancreatitis, or rare ophthalmologic concerns. Long-term safety data remain limited, particularly in older adults, pediatric patients, pregnant women, and individuals with comorbid disease. Emerging evidence also suggests potential roles in cancer prevention, hypertension, and neurodegenerative disease. As the indications for incretin-based therapies continue to expand, longer-duration trials and more diverse study populations will be essential to fully examine their long-term clinical effects. Though these agents hold considerable promise, careful and informed use remain vital.

## Introduction

Obesity continues to be a pressing concern nationwide, representing one of the most significant public health challenges in the modern era. Despite widespread implementation of structured exercise programs, carefully regulated diet plans, professional nutritional assistance, and bariatric surgery, obesity rates remain alarmingly high. At the national level, the prevalence of obesity among adults has continued to increase, rising by roughly 25.7% in males and 47.9% in females between the years of 1990 and 2021 [1]. A recent study, analyzing pre-2020 trends, prior to the widespread uptake of new anti-obesity pharmacotherapies in the US, estimated that the number of adults with obesity will grow from 107 million (42.9% of the US adult population) in 2023 to 138 million (50.5%) in 2060 [2]. These findings underscore the limited effectiveness in conventional lifestyle interventions and previously available pharmacological treatments in combating obesity. This continued to be the case until the advent of GLP-1 (glucagon-like peptide-1) and GIP (glucose-dependent insulinotropic polypeptide) analogues, novel therapeutic agents that have revolutionized the landscape of obesity management. GLP-1 and GIP are two naturally occurring incretin hormones produced in the gastrointestinal tract that play a vital role in glucose metabolism. Both act on pancreatic cells to regulate blood glucose levels following food intake [3,4]. GLP-1 is a peptide hormone primarily secreted by enteroendocrine L-cells located in the distal ileum and colon, as well as in certain neurons of the brainstem and a subset of pancreatic alpha cells [5]. It is released in response to nutrient ingestion and binds to G protein-coupled GLP-1 receptors. Activation of these receptors stimulates insulin secretion from pancreatic beta cells and suppresses glucagon release from alpha cells, both of which help lower blood glucose levels [5]. On the other hand, GIP is secreted from K-cells located in the duodenum and jejunum, increasing insulin in a glucose dependent manner as well, working synergistically with GLP-1. However, in contrast to GLP-1, GIP has a positive effect on glucagon release, increasing glucose levels [6]. The pancreas contains clusters of endocrine cells known as the Islets of Langerhans, composed primarily of alpha cells, which release glucagon, and beta cells, which secrete insulin through cAMP- and calcium-mediated pathways. When glucose is absorbed orally, it triggers what is known as the incretin effect, a phenomenon describing how oral glucose ingestion leads to the release of GLP-1 and GIP [7]. These incretins enhance insulin secretion, promoting better glucose control compared to when glucose is administered intravenously.

Beyond these pancreatic effects, incretins have been found to influence pathways outside the pancreas, including central nervous system circuits that regulate appetite and satiety, as well as peripheral mechanisms that handle energy balance, such as adipose tissue metabolism and overall energy expenditure [6]. These multifaceted actions highlight the potential of targeting incretin pathways for metabolic disease. Building on this physiological understanding, pharmaceutical therapies were developed to mimic incretin activity, initially aimed at improving glycemic control in individuals with type 2 diabetes. Clinical observations soon revealed that these agents also exerted profound effects on appetite, satiety, and weight, paving the way for the repurposing of incretin-based therapies, including dual GLP-1/GIP receptor agonists, for weight management in both diabetic and non-diabetic patient populations.

### Extra pancreatic effects of incretins

Incretins have been shown to have influence on extra-pancreatic tissues, including the gastrointestinal tract, cardiovascular system, and the central nervous system. GLP-1 exerts several key inhibitory effects on gastrointestinal function. It inhibits both gastrin-induced and meal-induced gastric acid secretion. These effects are physiologically relevant, as physiological increases in plasma GLP-1 levels can elicit marked inhibition of gastric secretion, an effect that is established together with peptide YY (PYY), another L-cell–derived hormone. Moreover, GLP-1 can abolish acid secretion even during pure vagal stimulation, an effect that previously was found to only be lost after truncal vagotomy [8]. They also have been shown to delay gastric emptying, prolonging the time food remains in the stomach and upper intestinal tract after a meal, increasing the sensation of satiety [9]. These findings have been highly relevant for incretin-based therapies, as pharmacological GLP-1 receptor agonists can not only be beneficial in diabetes but also in obesity as they not only enhance glucose-dependent insulin secretion but also slow gastric emptying, reduce appetite, and decrease GI motility, thereby contributing to weight loss.

In addition to their gastrointestinal actions, incretins exert significant cardiovascular effects. GLP-1 receptors are present in cardiomyocytes, vascular endothelium, and smooth muscle cells. Activation of these receptors has been shown to promote endothelial function, decrease vascular inflammation, modulate oxidative stress, and enhance vasodilation, all of which help promote myocardial perfusion and coronary microvascular health. It has been shown that the activation of GLP-1 receptors can improve myocardial blood flow, increase microvascular blood volume, and improve perfusion reserve, especially in populations affected by diabetes and obesity who are at risk for microvascular disturbances [10,11]. In clinical studies, GLP-1 receptor agonists were found to lower the risk of major cardiovascular events by about 14%, including reductions in cardiovascular death, stroke, heart failure hospitalization, and overall mortality [12]. GLP-1 has the ability to also cross the blood-brain barrier, and thus is able to exert influence on the central nervous system as receptors have been found in many parts of the brain. One of the main places where the body makes GLP-1 is a group of neurons in the brainstem, specifically in the nucleus of the solitary tract (NTS) and partly in the area postrema, together called the dorsal vagal complex [13]. These neurons also appear in other areas like the olfactory bulb, parts of the medulla, and the lower spinal cord. The NTS GLP-1 neurons become active when they receive signals from the vagus nerve. These signals communicate to the brain that the stomach is stretching indicate satiety or hormones released after a meal like GLP-1, cholecystokinin, and leptin are being released [13]. These neurons essentially are involved in brain regions that play a role in managing appetite, stress, reward, memory, and autonomic functions.

### GLP-1 and GIP-directed therapies

**Figure 1:**
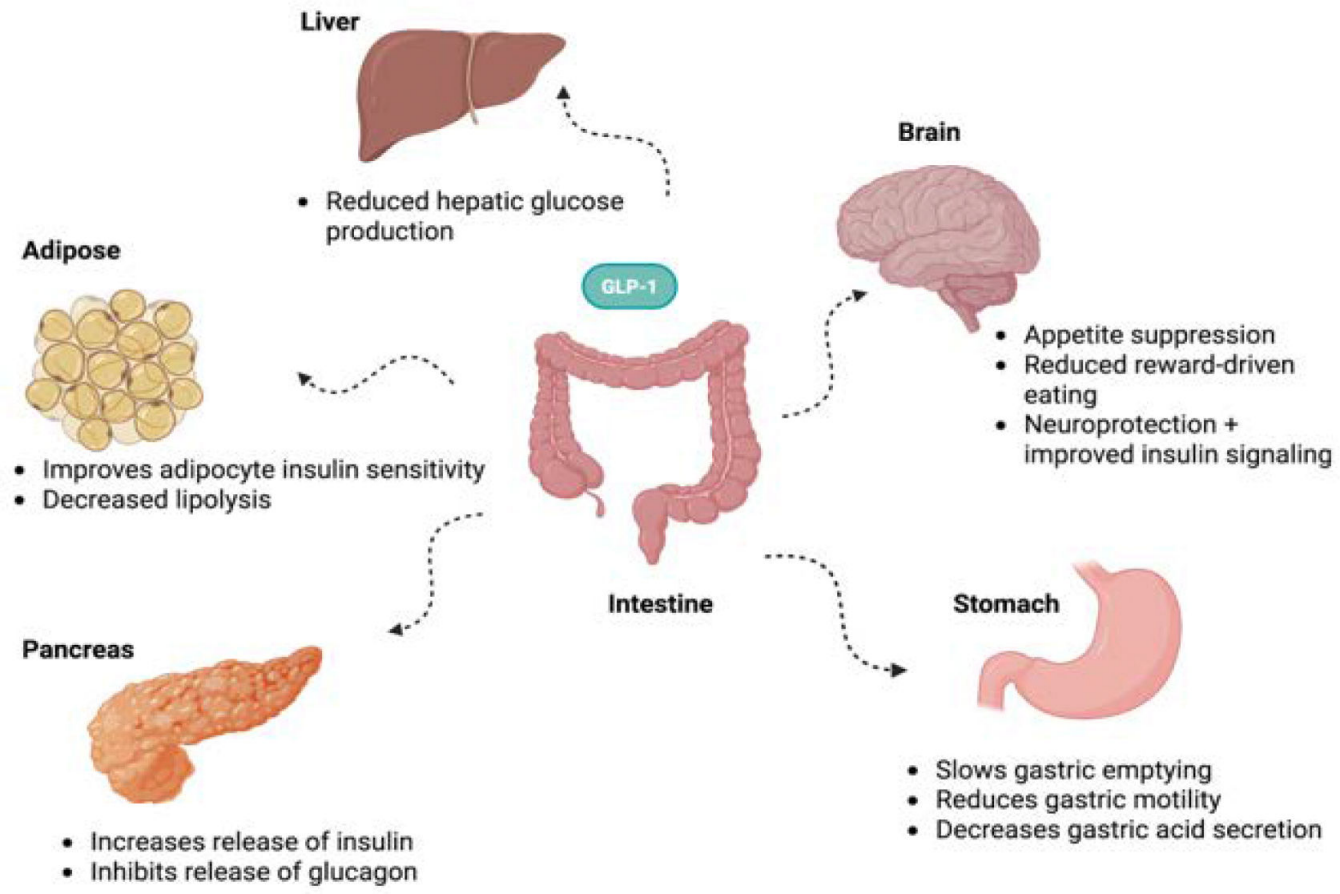
This illustration depicts how GLP-1 acts on multiple organs, including the stomach, pancreas, liver, adipose tissue, and brain to regulate metabolism, appetite, and hormone secretion. These coordinated actions help improve glucose control and reduce inflammation. Additionally, GLP-1 supports overall weight loss and enhances feelings of satiety

GLP-1 receptor agonists and GLP-1/GIP dual agonists have revolutionized the management of type 2 diabetes and obesity by targeting multiple pathophysiologic mechanisms (Figure 1).

Semaglutide is a long-acting GLP-1 receptor agonist with structural modifications that increase its affinity for the GLP-1 receptor and reduce degradation by DPP-4, thereby prolonging its availability in the body. It mimics the effects of endogenous GLP-1 by binding to receptors on pancreatic beta cells to enhance glucose-dependent insulin secretion and inhibit glucagon release. Semaglutide also slows gastric emptying and reduces appetite by activating receptors in the hypothalamus and brainstem, promoting satiety and decreasing caloric intake [14]. Semaglutide is available in two main formulations with distinct clinical indications. Ozempic, one formulation of semaglutide is approved for adults with type 2 diabetes to improve glycemic control and for those with type 2 diabetes and established cardiovascular disease to reduce the risk of major adverse cardiovascular events (MACE), including cardiovascular death, nonfatal myocardial infarction, and nonfatal stroke [15]. It is administered as a once-weekly subcutaneous injection, starting at 0.25 mg weekly for 4 weeks and titrated to maximum of 2 mg [16]. Wegovy, the other formulation, is approved for chronic weight management in adults with obesity (BMI ≥30 kg/m^2^) or overweight (BMI ≥27 kg/m^2^) with at least one weight-related comorbidity, such as hypertension, dyslipidemia, or type 2 diabetes. It is administered as a once-weekly subcutaneous injection via a prefilled pen, starting at 0.25 mg/week and gradually titrated every four weeks to a maximum dose of 2.4 mg/week [17].

Clinically, semaglutide has demonstrated potent weight-loss effects. In the STEP 4 trial, adults with overweight or obesity who continued weekly subcutaneous semaglutide 2.4 mg for 68 weeks experienced an additional mean body weight loss of 7.9% from week 20, compared with a 6.9% weight gain among those switched to placebo, representing a total difference of 14.8%. Waist circumference also decreased by 9.7 cm in the semaglutide group versus placebo, highlighting the efficacy of this therapy for sustained weight reduction. In addition to weight management, semaglutide significantly reduces the risk of type 2 diabetes. Among participants completing a 20-week run-in period, the baseline estimated 10-year diabetes risk fell from approximately 20.6% to 11.1% [18]. With continued treatment to week 68, the projected risk further declined to 7.7%, suggesting that semaglutide not only promotes weight loss and improves glycemic control, but may also substantially delay or prevent the onset of type 2 diabetes in high-risk populations [19]. These effects are likely mediated through a combination of weight loss, improved insulin sensitivity, and direct pancreatic effects, making semaglutide a powerful agent in diabetes prevention strategies.

**Figure 2:**
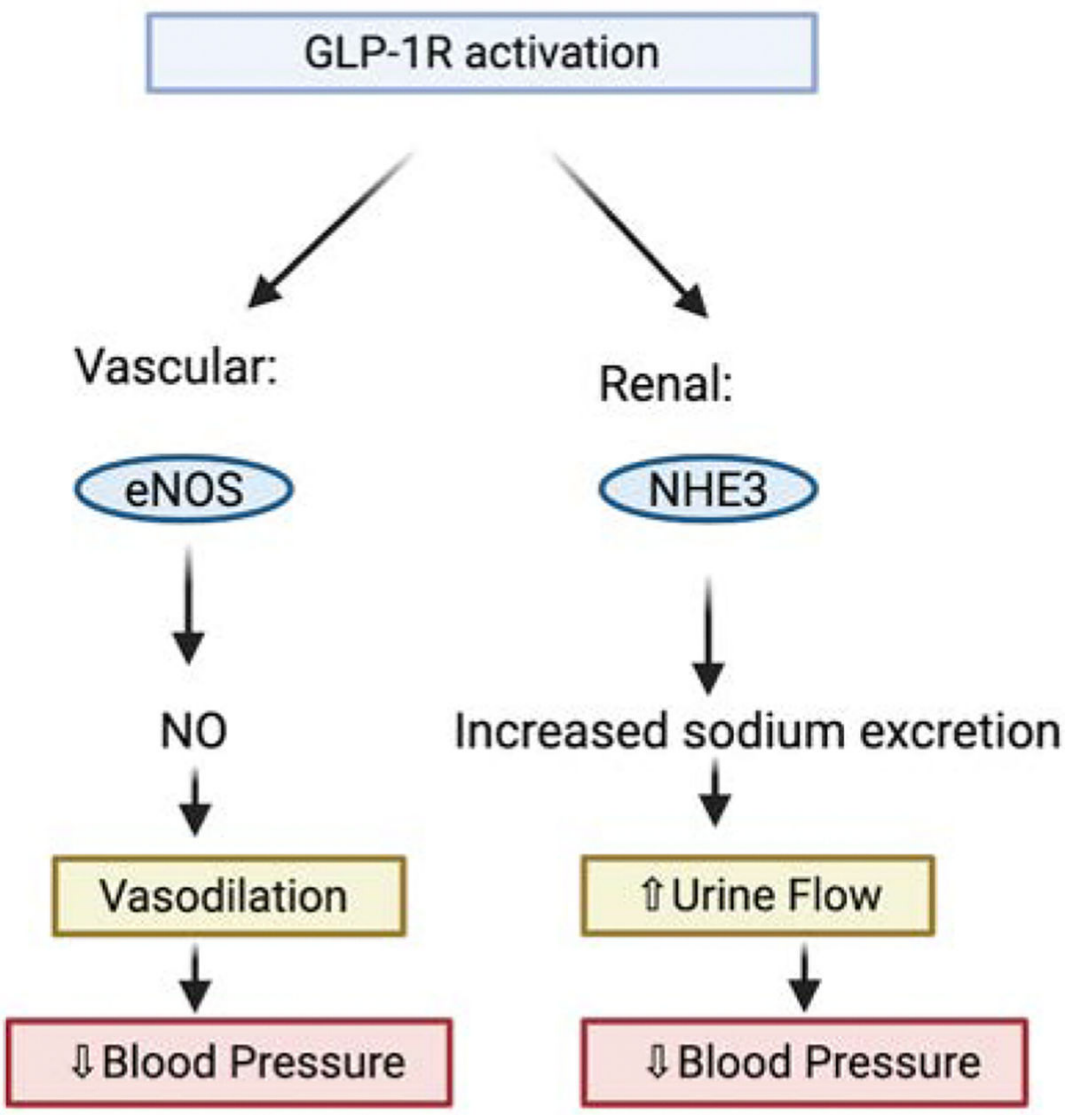
This figure demonstrates how GLP-1 receptor activation lowers blood pressure through two vital pathways: vascular eNOS-mediated nitric oxide production leading to vasodilation, and renal NHE3 inhibition resulting in increased sodium excretion and urine flow. Both mechanisms join to create a reduction in blood pressure.

GLP-1 receptor agonists have been associated with reductions in MACE and improvements in blood pressure, diuresis, lipid profiles, and markers of inflammation (Figure 2). Several cardiovascular outcomes trials (CVOTs) in patients with overweight or obesity, have demonstrated a reduced risk of atherosclerotic cardiovascular events with GLP-1 receptor agonists [20]. The SELECT trial, a randomized, double-blind, placebo-controlled study, evaluated semaglutide as an adjunct to standard care for the prevention of MACE in patients with established cardiovascular disease and overweight or obesity. SELECT demonstrated that once-weekly subcutaneous semaglutide was associated with a decreased risk of MACE, comprising cardiovascular death, nonfatal myocardial infarction, and stroke, compared with placebo, even in patients without diabetes [21,22].

**Figure 3:**
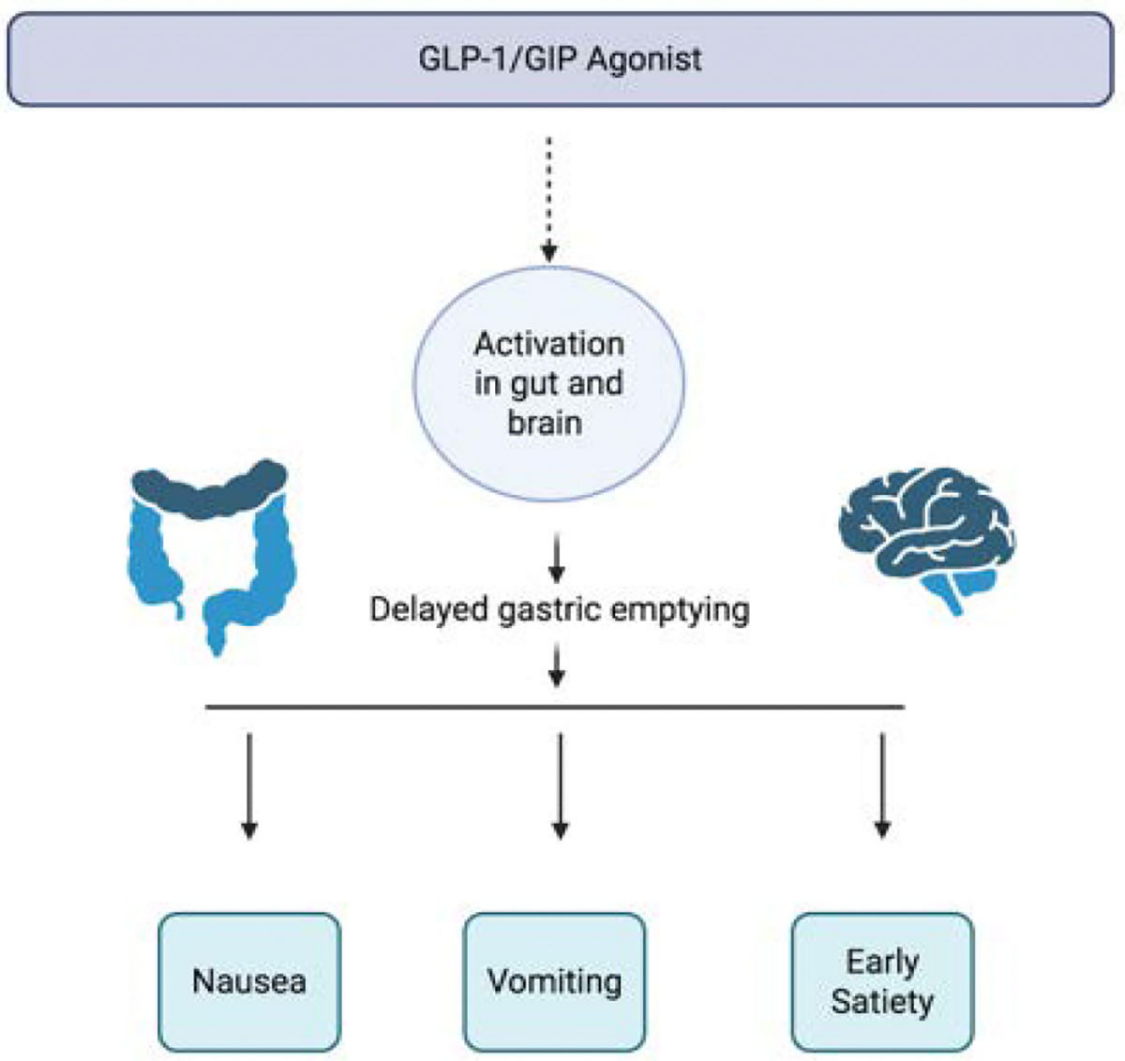
This diagram shows how GLP-1 and GIP agonists activate receptors in both the gastrointestinal tract and brain. These medications produce a notable delay in gastric emptying. As a result, common gastrointestinal effects such as nausea, vomiting, and early satiety can occur.

Tirzepatide is a novel dual GLP-1/GIP receptor agonist that simultaneously targets both incretin pathways, offering synergistic effects on glucose homeostasis, energy balance, and regulation of hunger (Figure 3). Similar to semaglutide, tirzepatide is available in two forms depending on the indication for use. Monjaro is approved for type 2 diabetes to improve glycemic control whereas Zepbound is approved for chronic weight management. Both are administered once weekly via subcutaneous injection and the recommended starting dose is 2.5 mg weekly, up to a maximum dose of 15 mg. By activating GLP-1 and GIP receptors, tirzepatide not only enhances glucose-dependent insulin secretion and suppresses glucagon but also promotes significant satiety and reduced caloric intake, similar to GLP-1 monotherapy, while also augmenting beta-cell function through the GIP-mediated pathway. In the present trial, adults with obesity had average weight reductions of 19.5% and 20.9% with 10-mg and 15-mg doses of tirzepatide, respectively, as compared with a 3.1% weight reduction with placebo [23]. In the SURMOUNT-5 trial, adults with obesity treated with tirzepatide achieved mean body weight reductions of up to 20.9% with the 15 mg dose, compared with 13.9% with semaglutide 2.4 mg, and only minimal reductions with placebo. Tirzepatide also led to greater decreases in waist circumference and visceral adiposity, suggesting pronounced effects on central fat distribution and cardiometabolic risk [24].

Emerging evidence indicates that tirzepatide may offer cardiometabolic advantages comparable to GLP-1 receptor agonists. Improvements in blood pressure, lipid profiles, and markers of inflammation have been observed, though long-term cardiovascular outcomes trials are ongoing to fully define its effect on MACE. Early data suggest that, like semaglutide, tirzepatide could provide meaningful reductions in cardiovascular risk among high-risk populations. Clinical trials, such as the SURPASS program, have shown reductions in systolic and diastolic blood pressure as well as improvement in LDL, cholesterol, and triglycerides in individuals with diabetes or obesity [22,25]. Additionally inflammatory markers such as CRP, IL-6, and TNF-α were shown to be suppressed, lowering likelihood of metabolic risk [26]. Its improvement in insulin resistance and hepatic lipid metabolism along with inflammation have served significant benefits in reducing risk of progression of atherosclerosis.

### Adverse effects of GLP-1 and GIP-directed therapies

Many of the adverse effects associated with GLP-1 and GIP-directed therapies are shared across the class (Table 1). However, there are several notable differences that exist between the individual agents. Wegovy and Ozempic, both of which contain semaglutide, exhibit comparable side-effect profiles. Despite such similarities, they may differ in regard to their adverse effects. Side-effects of such medications may be more frequent or more pronounced with Wegovy due to its higher maximum dose of 2.4 mg compared with 2 mg for Ozempic.

Semaglutide has been associated with significant gastrointestinal disturbances, including nausea, vomiting, and diarrhea, common effects for this drug class. When compared with placebo, subcutaneous semaglutide for 30 weeks in adults with type 2 diabetes mellitus induced nausea in 11.4 to 20% of the semaglutide-treated patients (placebo 3.3–8%), vomiting in 4 to 11.5% (placebo 2–3%) and diarrhea in 4.5 to 11.3% (placebo 1.5–6%). Additionally, higher doses of the drug as well as faster dosage incremental increases have been shown to have greater GI adverse effects than lower doses. One study showed how 77% of patients experienced GI adverse effects when a fast 2-week dose escalation was used to reach 40 mg compared with 54% in the slower 8-week dose-escalation group [27]. Rare cases of acute pancreatitis, hypoglycemia, acute kidney injury, diabetic retinopathy in patients with type 2 diabetes, angioedema and anaphylaxis have been reported with semaglutide. Mild acute pancreatitis was reported in three participants in the semaglutide group with type two diabetes mellitus (one participant had a history of acute pancreatitis, and the other two participants had both gallstones and pancreatitis); all recovered during the trial period. The mechanism of this is due to activation of GLP-1 receptors on pancreatic acinar cells which may enhance exocrine enzyme synthesis and secretion, potentially predisposing to autodigestion if enzymes are prematurely activated. Alterations in pancreatic ductal function and fluid flow may contribute to stasis, further increasing the risk of local inflammation. Indirectly, the efefcts of semaglutide on gastric emptying and biliary motility may increase the risk of gallstone formation, which is a common cause of secondary pancreatitis [28]. Additionally, many patients exhibit asymptomatic elevations in serum amylase and lipase, reflecting increased exocrine activity or minor subclinical pancreatic stress, although these elevations rarely progress to clinically significant pancreatitis (Table 1).

Rare cases of gastroparesis have been reported with semaglutide consumption (Table 1). For example, a case study describes a patient who started taking Semaglutide and subsequently developed symptoms consistent with gastroparesis namely persistent nausea, bloating, early satiety (feeling full very quickly), and abdominal discomfort. The authors note that while semaglutide is frequently well tolerated, this case illustrates that in some circumstances it may unmask or trigger a delayed-gastric-emptying syndrome [29].

As for more serious complications, such as malignant neoplasms, there was no difference between groups. Some semaglutide formulations carry a boxed warning for thyroid C-cell tumors in the U.S., based solely on rodent studies. In rodents, thyroid C-cells, which produce calcitonin, express high levels of GLP-1 receptors. Activation of these receptors increases calcitonin production, causes C-cell hyperplasia, and raises the risk of medullary thyroid tumors. Early studies suggested GLP-1 receptors were present in human thyroid tissue and medullary thyroid carcinoma, but these findings were later disproven due to unreliable testing methods [30].

In the treatment of obesity or overweight, the majority of these GI side effects seen in type two diabetics with Ozempic were considered mild-moderate and transient and did not require discontinuation. The majority of participants experienced the side effects within 20 weeks of starting semaglutide [31]. Furthermore, studies found an association between use of semaglutide and an increased risk of developing Non-arteritic Anterior Ischemic Optic Neuropathy (NAION), a rare but serious optic nerve disorder that could lead to blindness. Specifically, among patients with type 2 diabetes who were given semaglutide, the cumulative incidence of NAION over 36 months was about 8.9% versus ~1.8% in a comparator group not on GLP-1RAs. Among overweight or obese patients without diabetes treated with semaglutide, the incidence was ~6.7% vs ~0.8% in the comparator group over the same period [32]. However, the study does admit to be observational and thus cannot show causation. Semaglutide may also slightly increase heart rate, typically by 2–4 bpm, though the long-term clinical significance remains under investigation [33].

Tirzepatide, a dual GLP-1 and GIP receptor agonist, shares many class-wide adverse effects with other incretin-based therapies; however, its distinct mechanism of action and higher potency introduce several differences in tolerability (Table 1). Across clinical trials, gastrointestinal (GI) symptoms remain the most common adverse effects and tend to be dose-dependent, with higher doses (10–15 mg) associated with greater frequency and severity [34]. GI disturbances, including nausea, vomiting, and diarrhea, were reported at rates similar to or slightly higher than those observed with selective GLP-1 receptor agonists. In the SURPASS program, nausea occurred in approximately 12–24% of tirzepatide-treated patients (vs 6–12% with placebo or active comparators), vomiting in 5–14%, and diarrhea in 12–22% [35].

Regarding hypoglycemia, the risk remains low when tirzepatide is used without insulin or insulin secretagogue, similar to other GLP-1 receptor agonists. This is due to the fact that GLP-1 receptor agonists induce insulin secretion in a glucose dependent manner, reducing risk for hypoglycemia. In SURPASS-1 through SURPASS-3 and SURPASS J-mono, the rate of clinically significant hypoglycemia (blood glucose <54 mg/dL) ranged from 0% to 2%. In SURPASS-4, where 54% of participants were on sulfonylureas at baseline, hypoglycemia rates were higher (6–9%) but remained lower than those observed in the insulin glargine group (19%) [36]. This supports the overall low intrinsic hypoglycemia risk of tirzepatide when not combined with agents that independently induce insulin secretion.

On rarer but important adverse events, tirzepatide has been linked in RCTs to an increased risk of gallbladder/biliary disease, including cholelithiasis, although a clear dose–response relationship was not consistently seen. Data on cholelithiasis in patients with diabetes or obesity after treatment with tirzepatide were reported in the nine studies. The results showed that the risk of cholelithiasis in patients with diabetes or obesity was increased compared with that in the control group [37]. Tirzepatide activates both GLP-1 and GIP receptors; the drug may be associated with pancreatitis, at least theoretically in a similar mechanism to semaglutide. However, in a meta-analysis by Zeng et al. that included nine randomized controlled clinical trials, the increased risk of pancreatitis was not significantly associated with tirzepatide compared with all control groups consisting of basal insulin, selective GLP1-RAs, and placebo [38].

### Safety of semaglutide versus tirzepatide: direct comparison

In clinical studies of adults with obesity who do not have diabetes, both semaglutide and tirzepatide were associated with an increased risk of gastrointestinal (GI) adverse events compared with placebo. Overall, the likelihood of experiencing any GI side effect was nearly twice as high with either drug. Tirzepatide was associated with a higher risk than semaglutide, with a RR of 2.94 compared with 1.68 for semaglutide. The most commonly reported GI effects included nausea, diarrhea, vomiting, constipation, and abdominal discomfort, which were typically mild to moderate and most often occurred during the first several weeks of therapy [39].

Semaglutide, unlike tirzepatide, in obese individuals without diabetes was also linked to an increased risk of gallbladder-related problems, particularly cholelithiasis, with a more than 2.6-fold higher risk compared with placebo. Tirzepatide, in contrast, did not show a statistically significant increase in biliary complications in the available studies. Importantly, neither drug was associated with a significant increase in serious hepatic or pancreatic adverse events, including pancreatitis or liver injury, suggesting that severe organ toxicity is uncommon with these therapies [39].

Both drugs share some “class” risks for thyroid C-cell tumors in animal models, so are contraindicated for some patients with personal/family history of certain thyroid cancers. Both semaglutide has showed no increased risk in clinical studies as well as tirzepatide in which only preclinical, involving animal data for tirzepatide show dose-dependent thyroid C-cell hyperplasia or carcinoma in rodents. The meta-analysis doesn’t disprove those findings but shows absence of signal so far in human RCTs [40].

Both semaglutide and tirzepatide are associated with a low intrinsic risk of hypoglycemia. Both drugs are GLP-1 receptor agonists (tirzepatide is also a GIP agonist), which stimulate insulin secretion in a glucose-dependent manner, meaning insulin release is enhanced only when blood glucose is elevated. Clinical trials demonstrate that in patients not using concomitant insulin or insulin secretagogues, rates of clinically significant hypoglycemia (blood glucose <54 mg/dL) are extremely low. For instance, a safety analysis of tirzepatide, severe hypoglycemia was found to be extremely low at a rate of less than one percent across doses [41]. As for semaglutide, results from a meta-analysis suggested it did not increase the risk of hypoglycemia as compared to other therapies [42]. These findings suggest that both agents carry a low risk of hypoglycemia, provided they are not combined with other medications that independently stimulate insulin secretion, a caution that is particularly important in populations already affected by diabetes.

As for weight loss effectiveness, a meta-analysis of seven studies, including both randomized controlled trials and observational data, found that tirzepatide resulted in significantly greater weight loss than semaglutide. In this analysis, tirzepatide showed a standardized mean difference (SMD) of 0.75 compared with semaglutide, and at 6 months, it produced approximately 1.33% more weight loss [43]. It is important to note that this 6-month estimate reflects a pooled analysis across multiple studies and dosing regimens, rather than a single specific dose. Other evidence from clinical trials and network meta-analyses suggests a clear dose-dependent effect, with higher doses of tirzepatide (≥10 mg) producing more substantial weight loss, supporting the superior efficacy of tirzepatide over semaglutide in management of obesity or overweight [44]. This is likely in part due to additive effect of GIP receptor activation, which complements GLP-1–mediated improvements in insulin secretion, appetite suppression, and energy balance, supporting the enhanced weight-loss outcomes of tirzepatide over semaglutide in the management of obesity or overweight.

### Future implications of GLP-1/GIP receptor agonists

GLP-1 and GIP have shown promise in the realm of cancer prevention and treatments. These agents may have potential to evolve beyond their metabolic role in management of diabetes and obesity to serve as adjuncts in cancer prevention. This is the case especially for those cancers that are associated with obesity and metabolic disruption, including gastrointestinal, endometrial, and ovarian cancers [45]. This is due to the fact that the excess body fat is linked with heightened risk of developing cancer and worse prognosis in patients with these specific tumors due to several mechanisms. Obesity causes persistent, low-grade inflammation via dysfunctional adipose tissue as adipocytes release pro-inflammatory cytokines that promote the process of tumorigenesis [46]. Additionally, excess fat often leads to insulin resistance and hyperinsulinemia, and in turn increased insulin signaling drives cell proliferation [47].

Thus, treatment with GLP-1 and dual GLP-1/GIP agonists, which promote substantial weight loss, improve insulin sensitivity, and reduce systemic inflammation, may mitigate these cancer-promoting pathways. While more research is needed, emerging preclinical and epidemiologic evidence suggests that incretin-based therapies may play a meaningful role in reducing cancer risk and improving outcomes in obesity-associated malignancies.

For example, a retrospective study of 1,651,452 individuals with type 2 diabetes in the United States demonstrated that treatment with GLP-1 receptor agonists in comparison to insulin was associated with a decreased incidence in 10 of 13 obesity-associated cancers including pancreatic, colorectal cancer, ovarian cancer, and multiple myeloma [48]. In prostate cancer models, semaglutide decreased cell proliferation, glycolytic function, and phospho-kinase-mediated signaling [49]. Activation of the GLP-1 receptor in prostate tissue therefore appears to reduce cancer cell proliferation and slow the progression of prostate cancer.

Emerging data also suggest a potential role for GLP-1 receptor agonists in treating a wide range of conditions beyond obesity and type 2 diabetes, including hypertension and even neurodegenerative diseases such as dementia. Randomized controlled trials comparing incretin-based therapy with placebo and ≥ 1 month of follow-up have demonstrated that these agents produce a modest but clinically meaningful reduction in blood pressure and lower all-cause mortality in adults with overweight or obesity, without increasing the risk of hypoglycemia or pancreatitis [50]. Across studies, there was a significant association between the degree of weight loss and reductions in both systolic and diastolic blood pressure, highlighting weight-mediated and weight-independent cardiovascular benefits of GLP-1–based therapy.

The proposed mechanisms for blood pressure reduction include both vascular and renal effects. Vascularly, GLP-1 upregulates endothelial nitric oxide synthase (eNOS) activity and protein expression in endothelial cells via both GLP-1 receptor–dependent and GLP-1–related pathways, contributing to improved endothelial function, vasodilation, and overall vascular health [51]. Renally, GLP-1 induces diuresis and natriuresis. In a rat study, intravenous infusion of GLP-1 increased urine flow as well as the fractional excretion of sodium, potassium, and bicarbonate compared with vehicle-treated controls. These renal effects were accompanied by increases in renal plasma flow and glomerular filtration rate, highlighting a complementary mechanism by which GLP-1 receptor agonists may contribute to blood pressure reduction [52].

**Figure 4:**
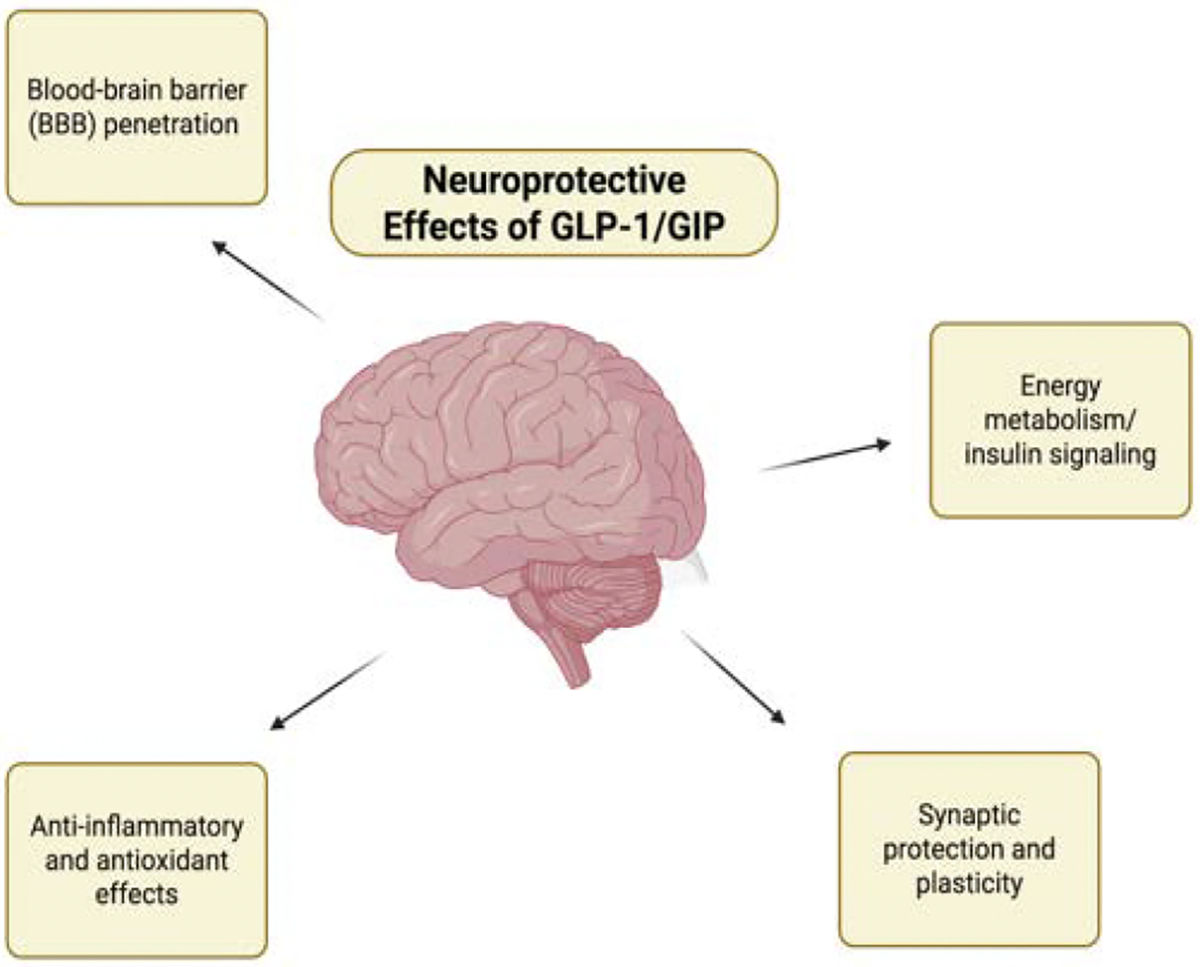
This figure illustrates the neuroprotective mechanisms attributed to GLP-1 and GIP. It highlights their ability to cross the blood-brain barrier and modulate key pathways involved in inflammation, oxidative stress, and energy metabolism. These incretin-based actions support synaptic protection and plasticity, contributing to overall brain health and resilience.

GLP-1 receptor agonists have also demonstrated significant neuroprotective potential in neurodegenerative conditions such as Parkinson’s disease and Alzheimer’s disease (Figure 4). In Alzheimer’s models, GLP-1R activation reduces amyloid-β deposition, tau hyperphosphorylation, and microglial neuroinflammation, while improving synaptic plasticity and neuronal insulin signaling [53]. These effects have been shown to enhance cognitive performance in multiple animal studies and early-phase human clinical trials. In Parkinson’s disease, GLP-1R activation preserves dopaminergic neurons in the substantia nigra by reducing oxidative stress and pro-inflammatory cytokines that contribute to neuronal destruction (Figure 4). By modulating central nervous system pathways, incretin-based therapies may therefore hold potential in preserving or improving cognitive function and slowing neurodegenerative disease progression [54].

### Limitations and areas for further investigation of GLP-1/GIP agonists

Despite their promising efficacy, there remains limited data regarding the long-term safety of GLP-1 and GIP receptor agonists. Most clinical trials and studies have been conducted over relatively short durations, typically less than ten years, leaving uncertainty about potential rare or adverse effects over extended use. Several reviews of the existing literature acknowledge that these therapies are relatively novel, and therefore the long-term consequences of their use over decades remain unknown and cannot yet be reliably predicted [55]. One area of potential concern is biliary or gallstone disease. Some data suggest that long-term use of GLP-1 receptor agonists, particularly at the higher doses that are used in management of obesity, may increase risk of biliary disease such as gallstones, cholelithiasis, a safety concern that can only be confirmed and supported prolonged use rather than in short-term trials [56].

Moreover, most studies of GLP-1 and GIP receptor agonists focus on their use in middle-aged adults with type 2 diabetes or obesity/overweight, leaving data in other populations, such as elderly individuals, pediatric patients, pregnant women, and those with comorbid conditions, limited. For instance, a prospective cohort study aggregating data from six Teratology Information Services identified 168 pregnancies with first-trimester GLP-1 receptor agonist exposure. The study found no increased risk of major birth defects compared with reference groups; however, higher rates of pregnancy loss and termination were observed in the exposure group. The authors noted that the small sample size and limited follow-up restricted the generalizability of their findings [57].

Furthermore, a review of existing literature highlighted that most clinical trials either do not fully describe participant characteristics or include very few older adults. In many trials, the proportion of participants aged over 75 years is exceedingly low, often less than 1% [58]. Given that older adults frequently have multiple comorbidities, the adverse effects and overall outcomes of these therapies may differ significantly from those observed in the typical middle-aged populations studied. Lastly, within the pediatric patient population, limited and short-term follow-up hinders the ability to know effects on growth and puberty and psychological development. A 2021 meta-analysis of RCTs in pediatric obesity showed modest weight changes and adjustments in BMI but did admit that long-term findings are beyond scope of the study as the trial durations were lacking extensive lengthy follow-up [59]. Another review of a total of 18 RCTs with 1402 participants with ages ranging from 6–17 years found that the median treatment duration was only 0.51 years, suggesting insufficient time to determine the long-term effects of these medications [60].

Furthermore, research on individuals with significant comorbid conditions remains limited. The data of the effectiveness of GLP-1/GIP medications is limited in patients with severely impaired kidney function, advanced heart failure, or unstable organ disease, meaning that safety risks and perceived benefits of such therapies in the sickest patients remains under-studied [61]. This is due to the fact that many of the noteworthy, randomized trials exclude individuals with very low eGFR or advanced or those with unstable or multiorgan pathology and so the effect of such medications in these patient populations is not as well-studied or understood [62]. There is a need for greater diversity in the populations studied to better understand the clinical effects of these therapies and to ensure that findings are more generalizable to real-world settings.

Long-term adherence and real-world tolerability are additional areas requiring investigation. Many trials involve intensive follow-up, frequent monitoring, and structured lifestyle counseling, which may not reflect real-world conditions [63]. In true clinical settings, patients may have several challenges that may influence their ability to maintain adherence with therapy, such as limited support systems in place, lack of educational or healthcare resources, and competing life responsibilities.

## Conclusion

GLP-1 and GIP receptor agonists represent a significant advancement in the management of obesity and type 2 diabetes. They provide meaningful improvements in glycemic control, weight reduction, and cardiovascular and neurological outcomes. However, these therapies are associated with notable adverse effects, including gastrointestinal (such as nausea, vomiting, and diarrhea), ophthalmological (such as visual disturbances and retinal toxicity), and, in rare cases, more severe outcomes such as organ toxicity or damage. As additional consequences of their use continue to be uncovered, the long-term safety profile of these medications remains incompletely understood. Expanding study populations to include more diverse age groups and clinical backgrounds, alongside longer-duration trials, will be essential to fully evaluate their risk-benefit profile. It is essential that the study of such therapies be conducted in a setting that closely aligns with real-world applications to maximize generalizability. Ultimately, GLP-1 and GIP agonists hold immense promise, and their use has potential extending beyond just treatment in obesity and type two diabetes. Nevertheless, cautious and informed use of these therapies is necessary. Continued research will be critical to maximize therapeutic benefits while minimizing potential harms.

## Figures and Tables

**Table 1:** Comparison of the adverse effect profiles of semaglutide and tirzepatide across key gastrointestinal and systemic symptoms. Nausea and vomiting remain the most frequently reported side effects for both agents. Worsening diabetic retinopathy emerging as a more recent concern.

Side Effect	Ozempic	Wegovy	Zepbound	Mounjaro
Gastrointestinal – Most Common Side Effect	Nausea, vomiting, constipation, abdominal pain	Same as Ozempic, higher incidence due to higher dosing	Nausea, vomiting, constipation, abdominal pain	Same as Zepbound, dose-dependent increase in GI symptoms
Hypoglycemia	Rare alone; higher risk with other glucose lowering agents	Same as Ozempic	Rare alone; slightly higher risk than semaglutide with other glucose lowering agents	Same as Zepbound
Pancreatitis	Rare	Same as Ozempic	Rare	Same as Zepbound
Gallbladder/gallstones	Possible	Same as Ozempic	Rare	Same as Zepbound
Thyroid C-cell tumor (animal studies)	Boxed warning: medullary thyroid carcinoma risk	Same as Ozempic	Boxed warning: medullary thyroid carcinoma risk	Same as Zepbound
Diabetic retinopathy	Worsening reported in T2D patients, with cases of blindness	Same as Ozempic	Worsening reported	Same as Zepbound
Injection-site reactions / fatigue / headache	Mild injection-site reactions, fatigue, headache	Same as Ozempic	Injection-site reactions, fatigue, headache, helching	Same as Zepbound

## Key Points

- Obesity rates continue to rise despite conventional lifestyle and pharmacologic interventions.
- GLP-1 and GIP incretin hormones regulate glucose homeostasis and influence appetite, weight, and energy balance.
- Semaglutide and tirzepatide produce substantial weight loss in both diabetic and non-diabetic populations.
- Tirzepatide consistently demonstrates greater weight reduction than semaglutide due to dual incretin receptor activity. This enhanced efficacy, however, was accompanied by a higher incidence of gastrointestinal adverse effects.
- GLP-1/GIP therapies provide cardiometabolic benefits, including improved blood pressure, lipid levels, and reduced cardiovascular events.
- Gastrointestinal adverse effects are the most common complications for both semaglutide and tirzepatide.
- Long-term safety data remain limited, particularly in elderly, pediatric, pregnant, and medically complex patients.
- Emerging evidence suggests potential roles in cancer prevention, neurodegenerative diseases, and hypertension.
- Increased risk of gallbladder disease is more clearly associated with GLP-1 monotherapy than dual agonists.
- Broader, longer-term, real-world studies are needed to fully assess safety and generalizability across diverse populations.
